# Comparative study on gut microbiota in three Anura frogs from a mountain stream

**DOI:** 10.1002/ece3.8854

**Published:** 2022-04-21

**Authors:** Zhuo Chen, Jun‐Qiong Chen, Yao Liu, Jie Zhang, Xiao‐Hong Chen, Yan‐Fu Qu

**Affiliations:** ^1^ College of Life Sciences Henan Normal University Xinxiang Henan China; ^2^ 12568 The Observation and Research Field Station of Taihang Mountain Forest Ecosystems of Henan Province Xinxiang Henan China; ^3^ 12534 Jiangsu Key Laboratory for Biodiversity and Biotechnology College of Life Sciences Nanjing Normal University Nanjing Jiangsu China; ^4^ College of Fisheries Henan Normal University Xinxiang Henan China

**Keywords:** Anura, bacterial similarity, bacterial transmission, gut microbiota, sympatric frogs

## Abstract

Composition and diversity in gut microbiota are impacted by a wide variety of factors. The similarity of gut microbiota in related or sympatric species has been gaining recent traction. Here, 16S rRNA gene sequencing technology was employed to study the gut microbiota of three sympatric frog species, namely *Odorrana tormota*, *O. graminea*, and *Amolops wuyiensis*. In these three frog species, the most abundant phylum was Proteobacteria, followed by Bacteroidetes, Verrucomicrobia, and Firmicutes. The most abundant family was Burkholderiaceae in three species. The most dominant genera were *Burkholderia*, *Caballeronia*, and *Paraburkholderia* with the highest relative abundance in *O. tormota*, *O. graminea*, and *A. wuyiensis*, respectively. No differences were observed in alpha diversity indexes among the three frog species. However, bacterial similarity of gut microbiota was significantly different between *O. tormota* and *A. wuyiensis* and between *O. graminea* and *A. wuyiensis*. Metabolism‐related gene function was predominantly enriched in the gut microbiota of the three evaluated frog species. From these findings, that the relative abundance of the gut microbiota and predicted gene functions differed in three species, we conclude that there were significant differences in the gut microbiota of the three species. Similar alpha diversity and interspecific bacterial similarity in the gut might be related to bacterial transmission among the three Anura frogs evaluated in this study.

## Graphical abstract

These three Anura frogs are commonly observed on rocks near streams. These species are distributed along mountain streams and congregate in large numbers during the breeding reason in mountain streams. This will provide a good model for the study on the gut microbial differences in sympatric frogs.

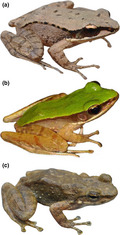

## 1 INTRODUCTION

Microbial composition and relative abundance in the gut microbiota have been studied in a wide variety of animals, such as mammals (Huang et al., 2021; Kartzinel et al., 2019), birds (Capunitan et al., 2020; Videvall et al., 2019), reptiles (Qu et al., 2020; Zhou et al., 2020), amphibians (Chang et al., 2016; Kohl et al., 2013), fishes (Li et al., 2014; Zhu et al., 2021), and many invertebrates (Chen et al., 2021). Amphibians are considered key species in the evolution of aquatic to terrestrial vertebrates (Takei, 2015). Moreover, most amphibians have complex life‐history traits across aquatic and terrestrial habitats (Petranka, 2007). Despite their evolutionary importance, research on the gut microbiota of amphibians is advancing at a slower pace compared to other vertebrate species, and only a limited number of studies have been carried out in a few species (Bletz et al., 2016; Chang et al., 2016; Huang et al., 2018; Kohl et al., 2013; Ya et al., 2019; Zhang, Gaughan, et al., 2019). Previous studies have shown that the dominant phyla in the gut microbiota of adult amphibians are Firmicutes, Bacteroidetes, and Proteobacteria (Huang et al., 2018) and that their diversity and relative abundance varied with life‐history stages and animal surroundings (Bletz et al., 2016; Zhang, Gaughan, et al., 2019). It is widely accepted that the gut microbiota plays an important role in nutrient absorption and digestion (Greer et al., 2016), vitamin biosynthesis (Huang et al., 2021), pathogen defense (Jing et al., 2020), and immune regulation (Dimitriu et al., 2013), thus affecting ecological adaptation (Kartzinel et al., 2019).

The composition and relative abundance of gut microbiota are influenced by multiple intrinsic and extrinsic factors and processes. Intrinsic factors can include host evolutionary status (Kartzinel et al., 2019), health status (Zeevi et al., 2019), and age (Videvall et al., 2019), whereas extrinsic factors include dietary habits (Tang et al., 2020), habitat (Zhang et al., 2018), and captivity (Zhou et al., 2020). For instance, the microbial relative abundance and associated function of the gut microbiota of two turtle species during long‐term domestication under the same conditions differed due to differences in genotypes (Qu et al., 2020). It has been shown that diversity in the gut microbiota of ostriches gradually increased with age and was impacted by cessation of yolk absorption (Videvall et al., 2019). Moreover, gut bacterial composition might differ within the same host species due to differences in microhabitat or dietary habits. For example, significant differences were found in the gut microbial composition of *Phrynocephalus vlangalii* living at different altitudes (Zhang et al., 2018). Moreover, gut bacterial composition and associated gene functions in laying hens were altered by heat stress (Zhu et al., 2019). Therefore, the impact of various factors on gut microbiota composition and relative abundance has been the research focus of many studies.

Gut microbiota is affected by environmental factors, including agricultural activity (Huang et al., 2018; Ya et al., 2019), habitat (Fontaine et al., 2018; Huang et al., 2018), dietary habits (Chang et al., 2016), host genotype (Kartzinel et al., 2019), age (Vences et al., 2016), and health status (Montalban‐Arques et al., 2015). Predicting the functions of the gut microbiota could contribute to the understanding of the physiological status and interspecific niche separation in hosts under different environmental conditions (Barelli et al., 2015; Huang et al., 2018). It has been demonstrated that high diversity in bacterial gene functions in farmland frogs correlated with diseases and pesticide degradation (Huang et al., 2018). In addition, several bacterial taxa (e.g., genus *Cellvibrio*) are known to contribute to adaptation to low temperatures and survival during hibernation in a terrestrial amphibian (*Plethodon cinereus*) (Fontaine et al., 2018). Therefore, functional changes in gut microbiota have an important effect on the health and survival of amphibians.

The relationship among sympatric species has been receiving considerable attention due to the potential gut bacterial transmission. The alpha and beta diversities of gut microbiota from *Bufo gargarizans* and other frog species were similar (Xu et al., 2020). Moreover, it has been shown that captive snakes (*Naja atra* and *Elaphe carinata*) share similar bacterial communities in the gut (Zhang et al., 2019). However, significant differences in the gut bacterial community structure were found among different frogs from a mountain area (Shu, Hong, Tang, et al., 2019). Therefore, comparing the gut microbiota of different amphibians is of great significance for understanding potential microbial transmission in sympatric species.

**FIGURE 1:**
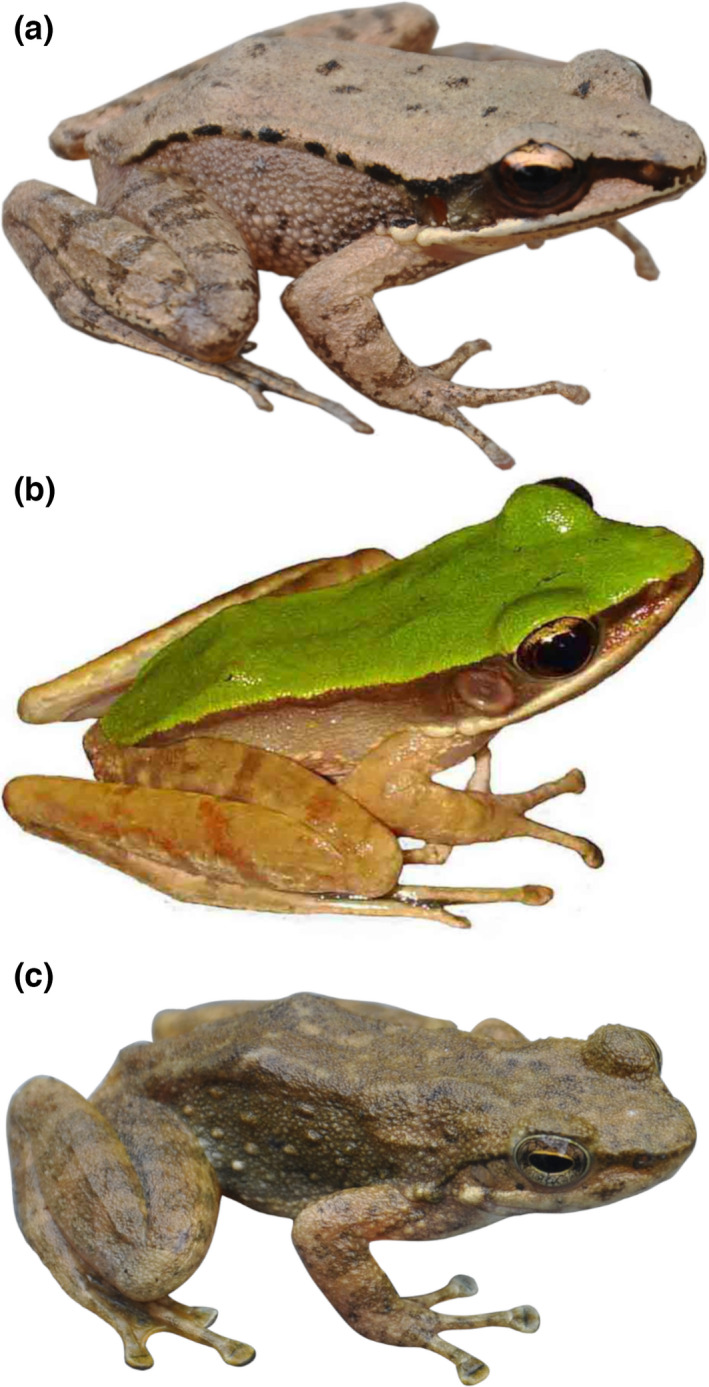
Photograph of the species studied (a) *Odorrana tormota*, (b) *Odorrana graminea*, and (c) *Amolops wuyiensis*

Here, we studied the gut microbiota of three Anura frogs, including *Odorrana tormota*, *O. graminea*, and *Amolops wuyiensis* (Figure 1), which commonly occur in southeast and central China (Fei et al., 2009). These species have been widely studied for their acoustic communication, morphology, molecular biology, and phylogeny (Chen et al., 2020; Fei et al., 2009; Feng et al., 2006; Shen et al., 2011). Although the microhabitats of these three species differ slightly (Fei et al., 2009), they are all commonly found on rocks near streams. These species are distributed along mountain streams and congregate in large numbers during the breeding reason in mountain streams. We can usually observe these three species in a transect less than 50 meters long in our collection site. This will provide a good model for the study on the gut microbial differences in sympatric frogs. Therefore, we would like to verify the potential of gut microbiome transmission in sympatric species by comparing the similarity of gut microbiota.

## 2 MATERIAL AND METHODS

### 2.1 Sample collection

A total of 21 adult male frogs from three species, namely *O. tormota* (*n* = 11), *O. graminea* (*n* = 7), and *A. wuyiensis* (*n* = 3) were collected in mid‐April 2020 within the same collection site at an altitude of 530 m in Huangshan, Anhui, Eastern China (30° 45’N, 118° 9’E). The collection site was a transect no more than 50 meters long along the stream which the water temperature was 14.7 ℃. We began to collect samples at 6:30 pm. Snout‐vent length (SVL) was 32.53 ± 0.27 mm in *O. tormota* (ranging from 31.02–33.79 mm), 49.98 ± 0.81 mm in *O. graminea* (ranging from 47.84–54.04 mm), and 42.24 ± 1.12 mm in *A. wuyiensis* (within the range of 39.63–46.57 mm). Frogs were euthanized immediately using 1% aqueous solution of tricaine methanesulfonate (MS‐222, Sigma‐Aldrich), and the intestinal tract of frogs was collected in full. The intestinal content was obtained by constriction and scraping under sterile conditions and transferred to a sterile centrifuge tube and stored at −80°C until further use for DNA extraction. The present study did not involve endangered or protected species. Experimental procedures adopted in the present study were approved by the Institutional Animal Care and Use Committee of Nanjing Normal University and were conducted in accordance with related guidelines (IACUC20200511).

### 2.2 DNA extraction and two‐step 16S rRNA gene sequencing

E.Z.N.A.^®^ stool DNA Kit (Omega Bio‐tek) was used to obtain total bacterial DNA according to manufacturer's instructions. DNA quantity and purity were measured using Qubit@3.0 (Thermo Scientific) and 1.0% agarose gel electrophoresis, respectively. Total DNA was used in PCR amplifications targeting the 16S rRNA V3–V4 genes using the universal primers 341F (5’‐CCTACGGGNGGCWGCAG‐ 3’) and 805R (5’‐GACTACHVGGGTATCTAATCC‐ 3’). PCR was performed in the reaction system with the total volume of 30‐μl, which consisted of 1 μl of forward primers, 1 μl of reverse primers, 15 μl of 2×Taq Master Mix, and 20 ng of genomic DNA. Thermocycling conditions were as follows: initial denaturation at 94°C for 3 min; followed by 5 cycles of denaturation at 94°C for 30 s, annealing at 45°C for 20 s, and extension at 65°C for 30 s; followed by a final extension at 72°C for 5 min. Subsequently, two unique 8‐base barcodes were introduced to both extremities of 16S rRNA amplicons. PCR amplifications were performed as follows: denaturation at 95°C for 3 min; followed by 5 cycles of 94°C for 20 s, 55°C for 20 s, and 72°C for 30 s; and final extension at 72°C for 5 min. PCR amplicons were obtained, purified, and quantified, and equivalent amounts of PCR amplicons were sequenced in the Illumina MiSeq platform using MiSeq Reagent Kit V3 (Illumina, Sangon Biotech Co., Ltd, Shanghai, China) according to manufacturer's instructions.

### 2.3 Data analysis

Raw DNA sequences were trimmed of primer sequences and paired‐end reads were merged using PEAR 0.9.6 (Zhang et al., 2014). Merged sequences were assigned to each sample according to their unique barcodes, disqualified reads were filtered out, and chimeric sequences were removed using DADA2 package (Callahan et al., 2016) and QIIME2 (Bolyen et al., 2019). Clean amplicon sequence variants (ASVs) were deposited in the National Genomics Data Center (NGDC) GSA database (accession number CRA005778). q2‐fragment‐classifier method in QIIME 2 against the Greengenes 13.5 database was used to assign each ASV to clusters into 97% similarity of operational taxonomic units (OTUs). To avoid large partial sample deviations, OTUs with the number greater than 10 in at least two samples were conserved for further analysis using QIIME2. Relative abundance of OTUs was standardized according to the sample with the least sequence number for further analysis.

MOTHUR 1.30.1 (Schloss et al., 2019) was used to calculate alpha‐diversity indexes, that is, community richness parameters (Chao1 index) and community diversity parameters (Shannon index), and data were processed in R v4.0 (R Development Core Team, 2020). Kruskal–Wallis test was conducted to compare differences in alpha diversity indexes among the three frog species. Principal component analysis (PCA) based on unweighted UniFrac distances was employed to determine beta‐diversity among the three frog species. Cluster analyses exploring similarities between gut microbial community composition from different frog species were performed using the analysis of similarity (ANOSIM) based on unweighted UniFrac distances. Linear discriminant analysis effect size (LEfSe) and linear discriminatory analysis (LDA) (Segata et al., 2011) were performed to compare microbial relative abundances at different taxonomic levels among different samples and evaluate the proportion of each level in the present study, only bacterial taxa with a logLDA score >3.5 and *p* < .05 were considered in analysis.

PICRUSt was conducted to query protein sequences and predict gene functions based on the Kyoto Encyclopedia of Genes and Genomes (KEGG) database (Kanehisa, 2019). Predicted gene functions were then assigned to the corresponding KEGG pathways (Langille et al., 2013) and the relative abundance in each pathway was determined. A Venn diagram was plotted to illustrate distribution of KOs genes among different frog species. LEfSe and LDA were used to compare the relative abundance of gene functions from KOs level 1–3 among the three frog species and only gene functions with log LDA score >2 were considered. All values are presented as mean ± standard deviation, and significance level was α = 0.05.

## 3 RESULTS

### 3.1 Gut microbiota characterization and operational taxonomic units (OTUs) classification

A total of 1,612,658 high‐quality reads were obtained from 1,754,959 raw reads from 21 samples. Rarefaction curves indicated that bacterial species richness and diversity stabilized as the number of sequences increased and are unbiased for each sample (Figure S1). A total of 2573 OTUs were identified at 97% sequence similarity, and each sample contained 145–775 OTUs (Table S1); OTUs were grouped into 24 phyla, 49 classes, 111 orders, 207 families, and 473 genera based on phylogenetic classification (Table S1).

### 3.2 Composition and abundance of gut microbiota in different species

**FIGURE 2:**
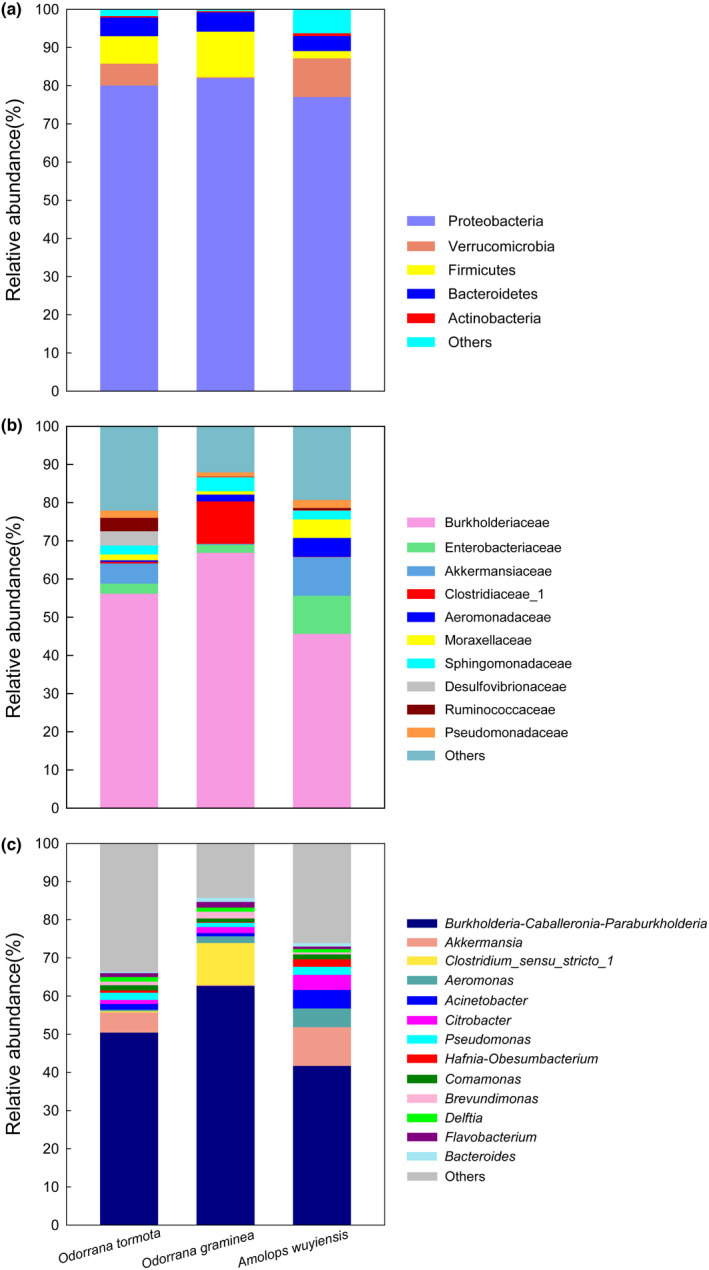
The relative abundance of the gut microbiota in three species at the phylum (a), family (b), and genus (c) levels. Each color in a plot represents a taxonomic group, of which the name is shown on the right side of the plot. The color for “others” indicates all other phyla (a), families (b), or genera (c) combined, of which the names are not listed in each plot

Proteobacteria, Bacteroidetes, Verrucomicrobia, and Firmicutes were the most abundant phyla (Figure 2a). Proteobacteria was the most dominant phylum in the gut microbiota of the three frog species, accounting for 91.10 ± 1.80%, 83.69 ± 9.79%, and 83.52 ± 9.69% in *O. tormota*, *O. graminea*, and *A. wuyiensis*, respectively (Figure 2a). The second most abundant phylum was Bacteroidetes in *O. tormota* (3.95 ± 0.76%), Firmicutes in *O. graminea* (11.37 ± 9.91%), and Verrucomicrobia in *A. wuyiensis* (8.29 ± 6.53%) (Figure 2a). The third most abundant phylum was Verrucomicrobia in *O. tormota* (3.60 ± 1.30%) and *O. graminea* (4.08 ± 1.40%), and Bacteroidetes in *A. wuyiensis* (4.23 ± 2.23%) (Figure 2a). The relative abundance of other phyla did not exceed 1% in all three species (Figure 2a).

Overall, 8 families were dominant in *O. tormota*, 9 in *O. graminea*, and 15 in *A. wuyiensis* with the relative abundance of over 1% (Figure 2b). Burkholderiaceae was the dominant family with the highest relative abundance among the three frog species, accounting for 56.48 ± 7.22% in *O. tormota*, 69.26 ± 10.21% in *O. graminea*, and 58.15 ± 17.92% in *A. wuyiensis* (Figure 2b). The dominant families with a relative abundance greater than 3% were Enterobacteriaceae (10.99 ± 2.82%), Moraxellaceae (5.52 ± 2.38%), Aeromonadaceae (5.49 ± 2.77%), and Akkermansiaceae (3.60 ± 1.30%) in *O. tormota*, Clostridiaceae_1 (10.82 ± 9.89%) and Sphingomonadaceae (3.67 ± 0.63%) in *O. graminea*, and Akkermansiaceae (8.29 ± 6.53%) and Enterobacteriaceae (3.80 ± 2.87%) in *A. wuyiensis* (Figure 2b).

At the genus level, *Burkholderia*, *Caballeronia* and *Paraburkholderia* were the genera with the highest relative abundance, accounting for 52.05 ± 7.49% in *O. tormota*, 65.00 ± 9.83% in *O. graminea*, and 51.81 ± 19.04% in *A. wuyiensis* (Figure 2c). Subdominant genera in *O. tormota* included *Acinetobacter* (5.52 ± 2.38%), *Aeromonas* (5.44 ± 2.74%), *Citrobacter* (4.61 ± 1.59%), and *Akkermansia* (3.60 ± 1.30%), whereas dominant genera in *O. graminea* and *A. wuyiensis* were *Clostridium sensu stricto 1* (10.71 ± 9.90%) and *Akkermansia* (8.29 ± 6.53%), respectively (Figure 2c).

### 3.3 Differences in gut microbiota composition among different species

**FIGURE 3:**
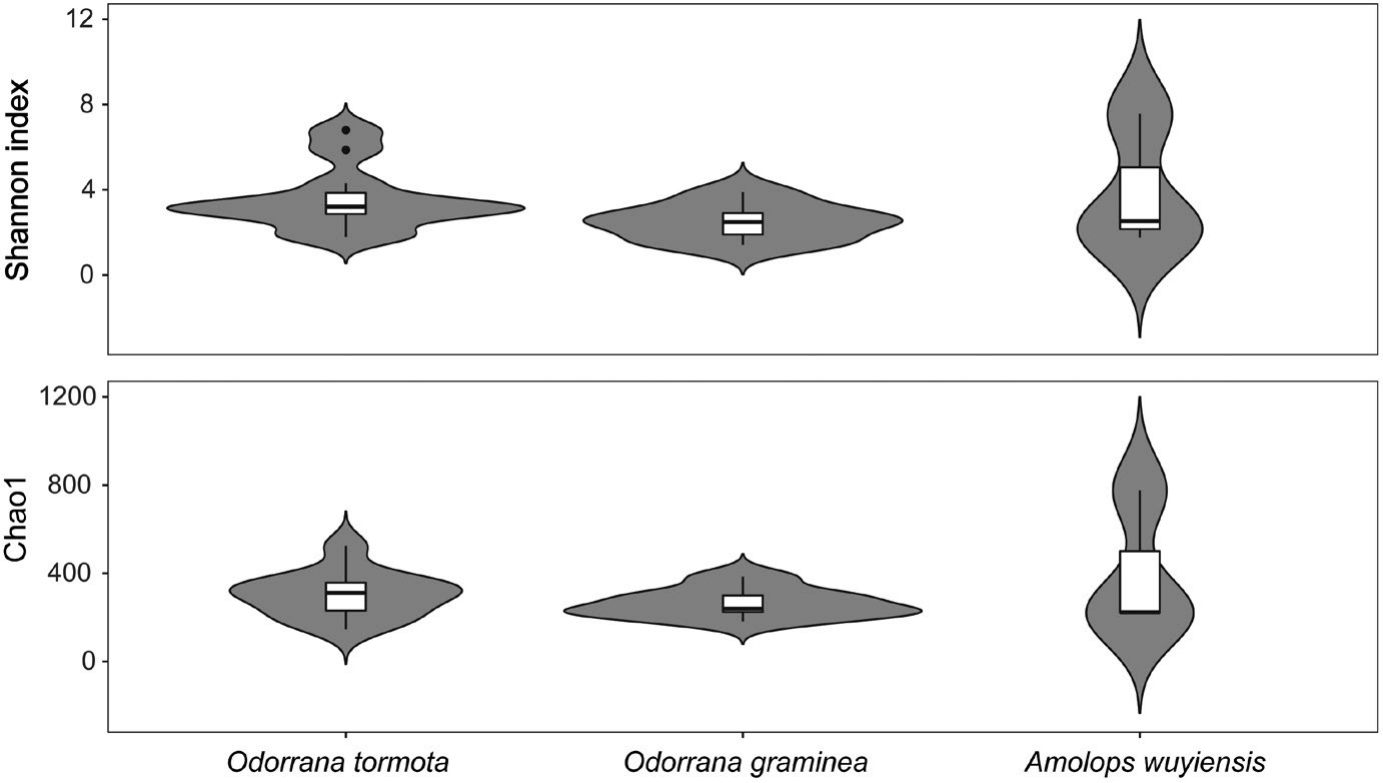
The alpha‐diversity indices of gut microbiota among the three species, including Shannon–Weiner and Chao1

**FIGURE 4:**
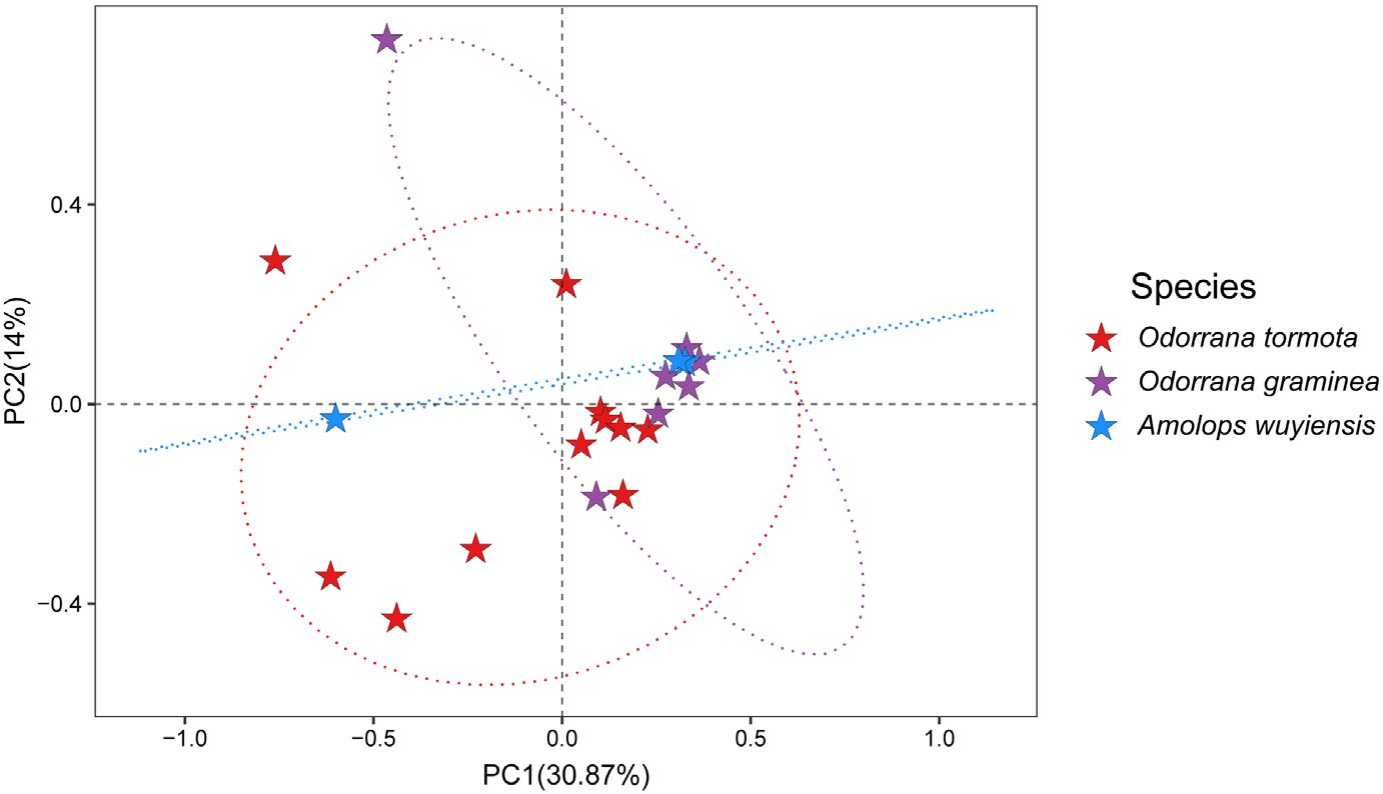
Gut microbiota diversity in the three species. Principal coordinates analysis of Bray–Curtis distance matrix for bacterial community diversity

The Kruskal–Wallis test showed that no significant differences in Shannon (*H* = 3.33, df = 2, *p* = .19) and Chao1 (*H* = 0.57, df = 2, *p* = .75) indexes were found among the three frog species (Figure 3). PCA showed no significant separation among the 21 samples with two first components (PCA1 and PCA2), accounting for 31% and 14% of the total variance in the samples, respectively (Figure 4). In contrast, ANOSIM analysis revealed significant differences in bacterial similarity among the three frog species (*R* = 0.30, *F*_2,18_ = 1.59, *p* = .01). Further analysis showed significant differences in gut bacterial composition between *O. tormota* and *O. graminea* (*R* = 0.33, *F*_1,16_ = 1.80, *p* = .009), but not between *O. tormota* and *A. wuyiensis* (*R* = 0.35, *F*_1,12_ = 1.57, *p* = .13) and between *O. graminea* and *A. wuyiensis* (*R* = 0.04, *F*_1,8_ = 1.26, *p* = .38).

**FIGURE 5:**
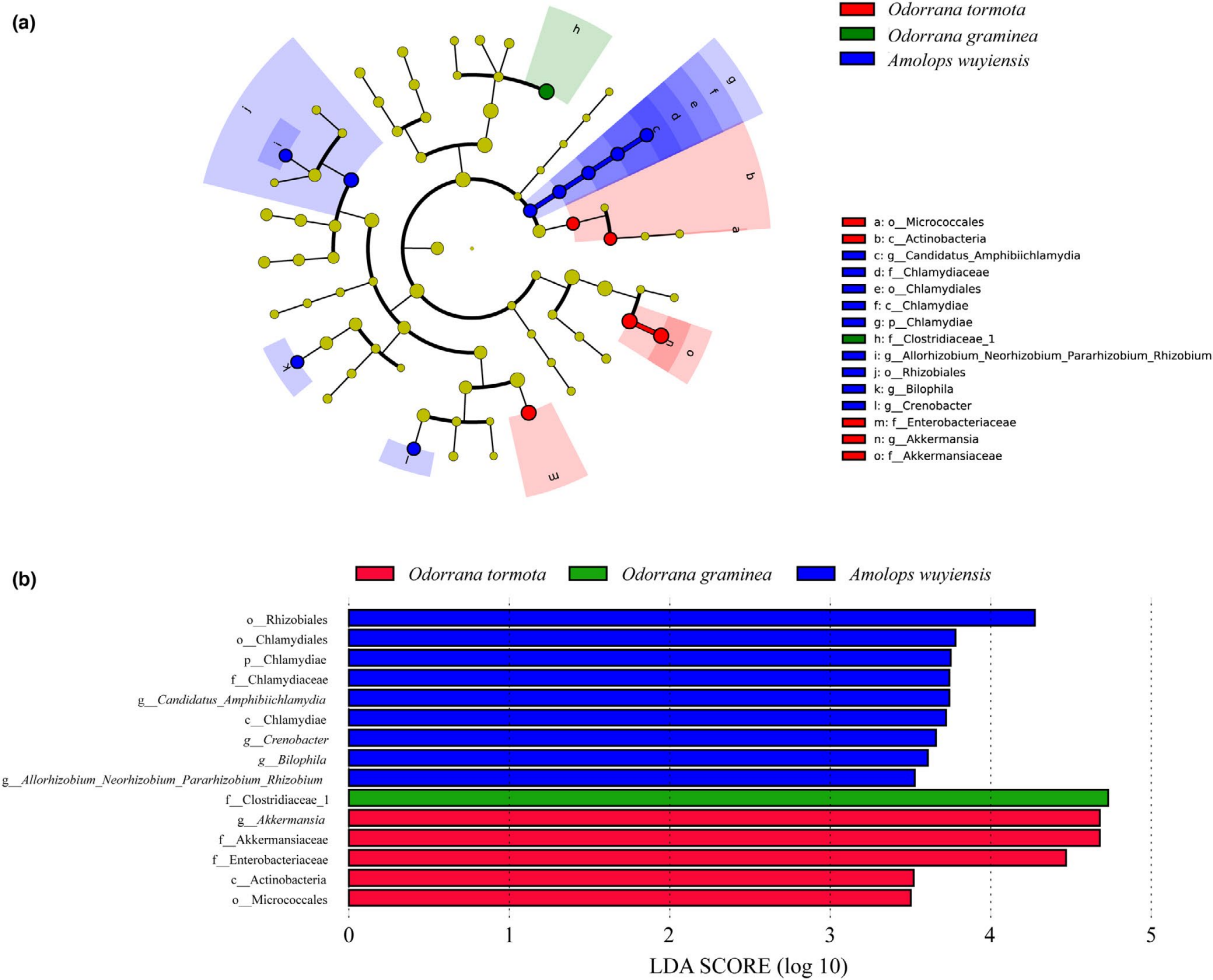
Linear discriminant analysis effect size (LEfSe) analysis of gut microbiota composition among the three species. Differences in bacterial taxa among the three species are determined by LEfSe (a). LDA scores reflect the differences in relative abundance among the three groups (b)

LEfSe analysis showed that there were significant differences in the gut bacterial taxon among the three species (Figure 5a). Considering bacterial taxa distribution, only bacteria in the lowest taxon among different species are listed. At the family level, LDA analysis showed that Clostridiaceae 1 (*LDA* = 4.73, *p* = .03) was present at greater proportion in *O. graminea* (Figure 5b). The relative abundance of Micrococcales (*LDA* = 3.50, *p* = .01) at the order level, *Enterobacteriaceae* (*LDA* = 4.47, *p* = .05) at the family level, and *Akkermansia* (*LDA* = 4.68, *p* = .03) at genus level was greater in *O. tormota* (Figure 5b). In *A. wuyiensis*, bacterial taxa with high relative abundance included *Candidatus amphibiichlamydia* (*LDA* = 3.74, *p* = .02), *Crenobacter* (*LDA* = 3.66, *p* = .01), *Bilophila* (*LDA* = 3.61, *p* = .01), and *Allorhizobium*‐*neorhizobium*‐*pararhizobium*‐*rhizobium* (*LDA* = 3.53, *p* = .02) at the genus level (Figure 5b).

### 3.4 Function prediction of gut microbiota

**FIGURE 6:**
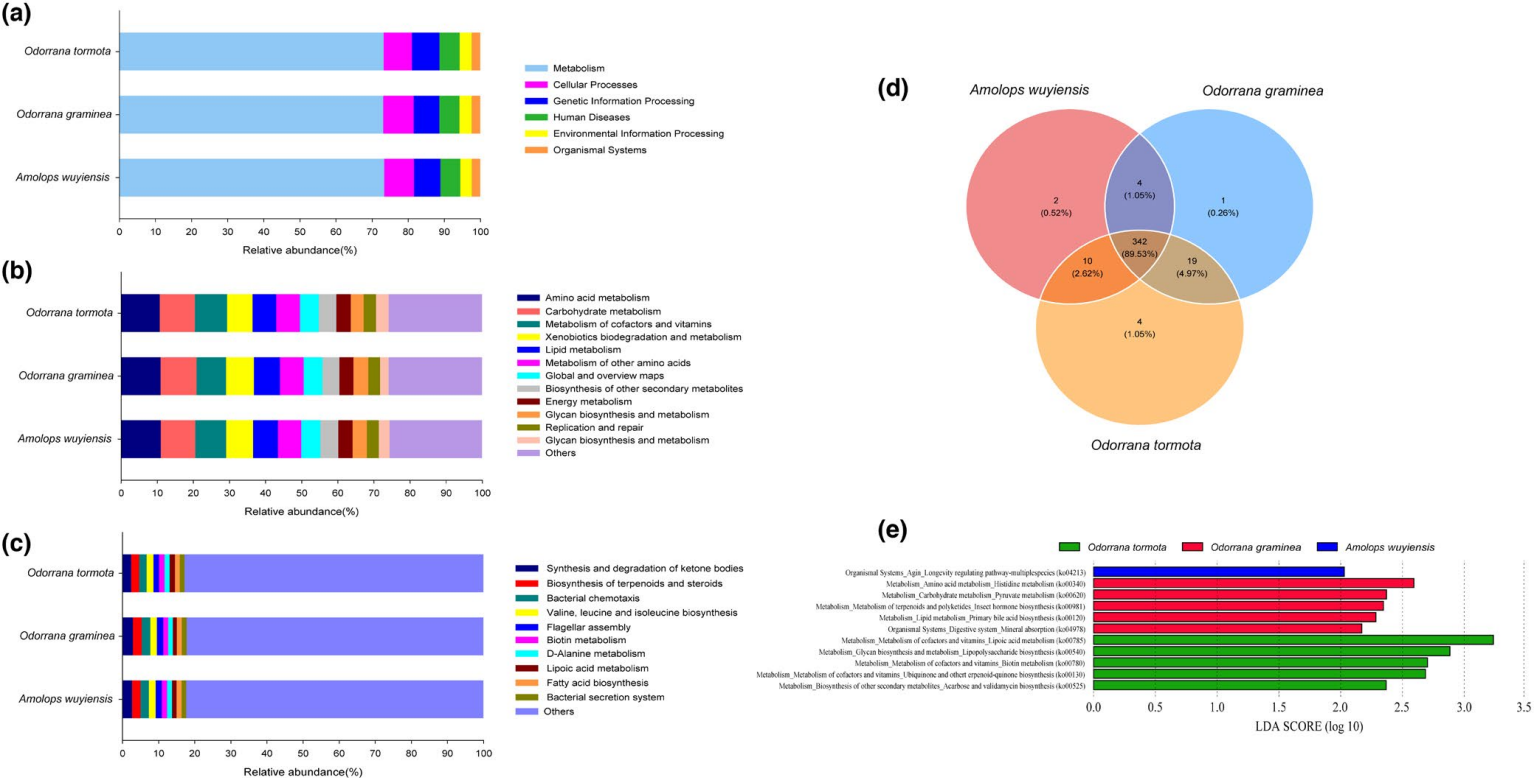
Gene functional categories based on 16S RNA in the gut microbiota at top (a), second (b) and third (c) levels of relative abundance, and Venn diagram of functional gene number of gut microbiota for three species (d). LDA scores reflect the differences in relative abundance of gene functions for three species (e). Each color in a plot indicates one gene function. Detailed descriptions are shown on the right side of each plot. The colors for others in Plots b and c indicate all other gene functions not listed in these two plots

The metabolism was the function with the highest relative abundance, accounting for 73.25 ± 0.39% in *O. tormota*, 73.08 ± 0.94% in *O. graminea*, and 73.38 ± 0.62% in *A. wuyiensis*, respectively (Figure 6a). Other highest‐ranked gene functions in descending order of relative abundance included cellular processes, genetic information processing, human diseases, environmental information processing, and organismal systems (Figure 6a). The relative abundance of gene function categories at the second enrichment level was related to metabolism, namely amino acid metabolism, carbohydrate metabolism, metabolism of cofactors and vitamins, xenobiotics biodegradation and metabolism, lipid metabolism, metabolism of other amino acids, and global/overview maps (Figure 6b). In addition, synthesis and degradation of ketone bodies, biosynthesis of terpenoids and steroids, and bacterial chemotaxis were the third‐level categories which accounted for over 2% of relative abundance in the gut microbiota of the three frog species (Figure 6c).

A total of 382 KOs were identified in the three frog species. Venn diagram of shared genes indicated that most KOs were shared among the different frog species (Figure 6d). LEfSe analysis based on KOs revealed evident differences in gene functions among the three frog species. At the third level, LDA discriminant analysis indicated that the longevity regulating pathway was significantly enriched in *A. wuyiensis* (ko04213; *LDA* = 2.03, *p* = .02). The histidine metabolism (ko00340; *LDA* = 2.59, *p* = .01), pyruvate metabolism (ko00620; *LDA* = 2.37, *p* = .04), insect hormone biosynthesis (ko00981; *LDA* = 2.35, *p* = .04), and primary bile acid biosynthesis (ko00120; *LDA* = 2.29, *p* = .01) related to metabolism as well as mineral absorption (ko04978; *LDA* = 2.17, *p* = .02) had the higher proportion in *O. graminea*. The lipoic acid metabolism (ko00785; *LDA* = 3.24, *p* = .02), lipopolysaccharide biosynthesis (ko00540; *LDA* = 2.88, *p* = .04), biotin metabolism (ko00780; *LDA* = 2.70, *p* = .01), ubiquinone and other terpenoid‐quinone biosynthesis (ko00130; *LDA* = 2.69, *p* = .004) and acarbose and validamycin biosynthesis (ko00525; *LDA* = 2. 73, *p* = .02) related to metabolism were enriched in *O. tormota* (Figure 6e).

## 4 DISCUSSION

In this study, although differences in the relative abundance of bacteria taxa and gene functions among three Anura frogs were significant, their gut microbial community remained highly similar (Figures 3, 4, 5). The most dominant phylum in the gut microbiota of the three frog species included in this study was Proteobacteria with a relative abundance of over 83% (Figure 2), which was consistent with the results of previous studies in other amphibians including *Rana dybowskii* (Tong et al., 2020), *R*. *amurensis* (Tong, Du, et al., 2019) and *Lithobates pipiens* (Kohl et al., 2013). Conversely, the most dominant phylum differed from another conspecific study conducted in other geographic regions (Shu, Hong, Tang, et al., 2019) and in other amphibians (Loudon et al., 2014; Tong, Du, et al., 2019; Weng et al., 2016). For instance, the dominant phylum in the gut microbiota of *Plethodon cinereus* was Verrucomicrobia (Loudon et al., 2014), and that of *Fejervarya limnocharis* was Firmicutes. Compared with another study carried out in the Banqiao Provincial Natural Reserve in China, the dominant phyla in the gut microbiota of *O. tormota* were Bacteroidetes (27%), Verrucomicrobia (24%), Firmicutes (23%), Fusobacteria (16%) and Proteobacteria (9%); the dominant phyla in the gut microbiota of *A. wuyiensis* were Firmicutes (45%), Proteobacteria (21%), Fusobacteria (21%), Bacteroidetes (7%), and Verrucomicrobia (3%) (Shu, Hong, Tang, et al., 2019). These results suggest that habitat significantly impacts gut microbiota composition in frogs, which has been demonstrated previously in *F*. *limnocharis* and *Babina adenopleura* collected in natural habitat and farmland sites (Huang et al., 2018). However, changes in gut microbiota composition within different habitats did not severely impact core physiological functions of gut microbiota (Huang et al., 2018).

Enrichment in the phylum Proteobacteria in gut microbiota is closely related to the habitat of evaluated animals. For example, Proteobacteria enriched in the gut of salamander larvae can be related to higher oxygen content in streams (Bletz et al., 2016). Bacteria in the phylum Proteobacteria are mainly correlated with catabolism and fermentation of complex sugars, and the potential biosynthesis of vitamins for the host (Colston & Jackson, 2016). The ratio of the relative abundance of Proteobacteria to the sum of the abundance of Firmicutes and Bacteroidetes phyla is used as a proxy to measure the tolerance of bacteria to cold environments, in which the higher the ratio, the stronger the tolerance (Cameron & McAllister, 2016). In this study, high abundance of the phylum Proteobacteria in the three frog species might be related to the low temperature (approximately 14°C) in the stream.

Bacteroidetes and Verrucomicrobia were the subdominant phyla in *O. tormota*, Firmicutes and Verrucomicrobia in *O. graminea*, and Verrucomicrobia and Firmicutes in *A. wuyiensis* (Figure 2). These bacteria largely contribute to maintenance of host healthy and are involved in energy absorption and metabolism. It is known that bacteria in the phylum Bacteroidetes can degrade complex macromolecules in order to facilitate their absorption by the host (Colston & Jackson, 2016). In contrast, bacteria within the phylum Firmicutes can produce enzymes involved in fermentation and vitamin B synthesis (Rowland et al., 2018). Finally, bacteria in the phylum Verrucomicrobia are potential (poly)saccharide degraders (He et al., 2017).

At different taxonomic levels, gut microbial composition in frogs can be significantly different due to multiple factors, including host genotype (Shu, Hong, Tang, et al., 2019), ontogenetic stage (Warne et al., 2019), gender (Shu, Hong, Yu, et al., 2019), habitat (Huang et al., 2018), and captivity (Tong, Liu, et al., 2019). Significant differences were found at the taxonomic level in the relative abundance of gut microbiota among the three frog species (Figure 5). A higher relative abundance of the order Micrococcales (Cui et al., 2018) was found in *O. tormota*. The genus *Bilophila* (Mohajeri et al., 2018) related to metabolism in *A. wuyiensis*. The genus *Akkermansia* is a biomarker for a healthy gut (Swidsinski et al., 2011) and could use mucus as a sole carbon and nitrogen source (Derrien et al., 2004) in *O. tormota*. However, a higher relative abundance of the Enterobacteriaceae family in *O. tormota* and Clostridiaceae 1 in *O. graminea* could be considered one of the major harmful microorganisms (Lupp et al., 2007; Zhou et al., 2015).

In this study, no significant differences were observed in alpha diversity of the gut microbiota in these three species (Figure 3). Alpha diversity is impacted by host genotype (Shu, Hong, Tang, et al., 2019), sampling source (Wu et al., 2019), habitat (Zhao et al., 2018), and ontogenetic stage (Videvall et al., 2019), whereas gut microbial community similarity is affected by factors such as health status (Bian et al., 2017) and dietary habits (Rojas et al., 2021). Increased gut microbial diversity in sympatric species might be associated with a potential intraspecies bacterial transmission. In this context, the host could acquire new gut bacteria from other animal species or conspecific individuals to maintain high species diversity in terrestrial vertebrates, such as pikas (Speakman et al., 2021) and lizards (Troyer, 1984). Likewise, sympatry can also increase intraspecific similarity in the gut microbiota, as observed in pikas and yaks in the Tibetan Plateau (Fu et al., 2021). Gut microbial transmission is beneficial for their hosts to broaden their dietary niches (Fu et al., 2021). Therefore, it can be hypothesized that a certain degree of microbial transmission occurs between *O. tormota* and *A. wuyiensis* and between *O. graminea* and *A. wuyiensis*, which resulted in close diversity and similarity of their corresponding gut microbiota. However, previous studies revealed significant differences in diversity and similarity between *O. tormota* and *A. wuyiensis* (Shu, Hong, Yu, et al., 2019). More studies are required to elucidate whether habitat and sampling time may have determined such differences.

In addition, it was demonstrated herein that the gene function most enriched in the gut microbiota of the three frog species was mainly metabolism with a relative abundance greater than 73% (Figure 6). This result is consistent with the function of gut microbiota in most amphibians, such as *R*. *dybowskii* (Tong et al., 2020) and *B*. *gargarizans* (Ya et al., 2019). Comparing with differences in the relative abundance of gene functions, the most enriched gene function in the gut microbiota of *O. tormota* was related to metabolism, including five different KO categories. Gene function with high relative abundance in *O. graminea* was correlated mainly with metabolism (four KO categories) and also with mineral absorption (1 KO category). In the gut microbiota of *A. wuyiensis*, only the KO related to longevity regulating pathway was enriched. Gene functional differences in the gut microbiota of the three frog species might be correlated with specific host metabolism due to dietary habits or other ecological factors.

## 5 CONCLUSIONS

Significant differences were found in the bacterial relative abundance and functions in the gut microbiota of the three Anura frogs evaluated in this study. Proteobacteria accounted for the highest proportion in the gut microbiota of the three frog species, which might be associated with metabolism and higher stress tolerance in cold streams. Similar alpha diversity and high interspecific bacterial community similarity in the gut microbiota were found between *O. tormota* and *A. wuyiensis* and between *O. graminea* and *A. wuyiensis*, which might be correlated with bacterial transmission among the three sympatric frog species.

## CONFLICT OF INTEREST

The authors declare that they have no competing interests for the publication of this study.

## AUTHOR CONTRIBUTION

**Chen Zhuo:**Conceptualization (lead); Data curation (supporting); Formal analysis (supporting); Funding acquisition (lead); Investigation (lead); Methodology (equal); Project administration (equal); Resources (lead); Software (lead); Supervision (lead); Validation (equal); Visualization (equal); Writing – original draft (equal); Writing – review & editing (equal). **Chen Jun‐Qiong:** Conceptualization (equal); Data curation (equal); Formal analysis (lead); Methodology (equal); Validation (equal); Visualization (equal); Writing – original draft (equal). **Liu Yao:** Data curation (equal); Investigation (lead); Methodology (equal); Resources (equal); Validation (equal); Writing – original draft (equal). **Zhang Jie:** Conceptualization (equal); Funding acquisition (equal); Investigation (supporting); Methodology (equal); Project administration (equal); Supervision (equal); Validation (equal); Writing – original draft (equal). **Chen Xiao‐Hong:** Data curation (supporting); Funding acquisition (equal); Investigation (equal); Methodology (equal); Project administration (equal); Resources (equal); Supervision (equal); Validation (equal); Writing – original draft (equal); Writing – review & editing (equal). **Yan‐Fu Qu:** Conceptualization (equal); Data curation (lead); Formal analysis (lead); Funding acquisition (equal); Methodology (equal); Project administration (equal); Resources (equal); Validation (equal); Visualization (lead); Writing – original draft (lead); Writing – review & editing (lead).

## Supporting information

Figure S1

Table S1

## Data Availability

Clean amplicon sequence variants (ASVs) were deposited in the National Genomics Data Center (NGDC) GSA database (accession number CRA005778).

## References

[ece38854-bib-0001] Barelli, C. , Albanese, D. , Donati, C. , Pindo, M. , Dallago, C. , Rovero, F. , Cavalieri, D. , Tuohy, K. M. , Hauffe, H. C. , & Filippo, C. D. (2015). Habitat fragmentation is associated to gut microbiota diversity of an endangered primate: implications for conservation. Scientific Reports, 5, 1–12. 10.1038/srep14862 PMC4595646 26445280

[ece38854-bib-0002] Bian, G. , Gloor, G. B. , Gong, A. H. , Jia, C. S. , Zhang, W. , Hu, J. , Zhang, H. , Zhang, Y. M. , Zhou, Z. Q. , Zhang, J. G. , Burton, J. P. , Reid, G. , Xiao, Y. L. , Zeng, Q. , Yang, K. P. , & Li, J. G. (2017). The gut microbiota of healthy aged chinese is similar to that of the healthy young. mSphere, 2(5), e00327‐17. 10.1128/mSphere.00327-17 28959739 PMC5615133

[ece38854-bib-0003] Bletz, M. C. , Goedbloed, D. J. , Sanchez, E. , Reinhardt, T. , Tebbe, C. C. , Bhuju, S. , Geffers, R. , Jarek, M. , Vences, M. , & Steinfartz, S. (2016). Amphibian gut microbiota shifts differentially in community structure but converges on habitat‐specific predicted functions. Nature Communications, 7, 13699. 10.1038/ncomms13699 PMC5171763 27976718

[ece38854-bib-0004] Bolyen, E. , Rideout, J. R. , Dillon, M. R. , Bokulich, N. A. , Abnet, C. C. , Al‐Ghalith, G. A. , Alexander, H. , Alm, E. J. , Arumugam, M. , Asnicar, F. , Bai, Y. , Bisanz, J. E. , Bittinger, K. , Brejnrod, A. , Brislawn, C. J. , Brown, C. T. , Callahan, B. J. , Caraballo‐Rodríguez, A. M. , Chase, J. , … Caporaso, J. G. (2019). Reproducible, interactive, scalable and extensible microbiome data science using QIIME 2. Nature Biotechnology, 37(8), 852–857. 10.1038/s41587-019-0252-6 PMC7015180 31341288

[ece38854-bib-0005] Callahan, B. J. , McMurdie, P. J. , Rosen, M. J. , Han, A. W. , Johnson, A. J. A. , & Holmes, S. P. (2016). DADA2: High‐resolution sample inference from Illumina amplicon data. Nature Methods, 13(7), 581–583. 10.1038/nmeth.3869 27214047 PMC4927377

[ece38854-bib-0006] Cameron, A. , & McAllister, T. A. (2016). Antimicrobial usage and resistance in beef production. Journal of Animal Science and Biotechnology, 7(1), 68. 10.1186/s40104-016-0127-3 27999667 PMC5154118

[ece38854-bib-0007] Capunitan, D. C. , Johnson, O. , Terrill, R. S. , & Hird, S. M. (2020). Evolutionary signal in the gut microbiomes of 74 bird species from Equatorial Guinea. Molecular Ecology, 29(4), 829–847. 10.1111/mec.15354 31943484

[ece38854-bib-0008] Chang, C. W. , Huang, B. H. , Lin, S. M. , Huang, C. L. , & Liao, P. C. (2016). Changes of diet and dominant intestinal microbes in farmland frogs. BMC Microbiology, 16, 33. 10.1186/s12866-016-0660-4 26966006 PMC4785643

[ece38854-bib-0009] Chen, L. , Li, S. X. , Xiao, Q. , Lin, Y. , Li, X. X. , Qu, Y. F. , Wu, G. G. , & Li, H. (2021). Composition and diversity of gut microbiota in *Pomacea canaliculata* in sexes and between developmental stages. BMC Microbiology, 21(1), 200. 10.1186/s12866-021-02259-2 34210255 PMC8252327

[ece38854-bib-0010] Chen, Z. , Li, H. Y. , Zhai, X. F. , Zhu, Y. J. , He, Y. X. , Wang, Q. Y. , Li, Z. , Jiang, J. P. , Xiong, R. C. , & Chen, X. H. (2020). Phylogeography, speciation and demographic history: Contrasting evidence from mitochondrial and nuclear markers of the Odorrana graminea sensu lato (Anura, Ranidae) in China. Molecular Phylogenetics and Evolution, 144, 106701. 10.1016/j.ympev.2019.106701 31811937

[ece38854-bib-0011] Colston, T. J. , & Jackson, C. R. (2016). Microbiome evolution along divergent branches of the vertebrate tree of life: what is known and unknown. Molecular Ecology, 25(16), 3776–3800. 10.1111/mec.13730 27297628

[ece38854-bib-0012] Cui, Y. X. , Fang, L. C. , Guo, X. B. , Wang, X. , Wang, Y. Q. , Li, P. F. , Zhang, Y. J. , & Zhang, X. C. (2018). Responses of soil microbial communities to nutrient limitation in the desert‐grassland ecological transition zone. Science of the Total Environment, 642, 45–55. 10.1016/j.scitotenv.2018.06.033 29894881

[ece38854-bib-0013] Derrien, M. , Vaughan, E. E. , Plugge, C. M. , & de Vos, W. M. (2004). Akkermansia muciniphila gen. nov., sp. nov., a human intestinal mucin‐degrading bacterium. International Journal of Systematic and Evolution Microbiology, 54, 1469–1476. 10.1099/ijs.0.02873-0 15388697

[ece38854-bib-0014] Dimitriu, P. A. , Boyce, G. , Samarakoon, A. , Hartmann, M. , Johnson, P. , & Mohn, W. W. (2013). Temporal stability of the mouse gut microbiota in relation to innate and adaptive immunity. Environmental Microbiology Reports, 5(2), 200–210. 10.1111/j.1758-2229.2012.00393.x 23584963

[ece38854-bib-0015] Fei, L. , Hu, S. Q. , Ye, C. Y. , & Huang, Y. Z. (2009). Anura. Science Press.

[ece38854-bib-0016] Feng, A. S. , Narins, P. M. , Xu, C. H. , Lin, W. Y. , Yu, Z. L. , Qiu, Q. , Xu, Z. M. , & Shen, J. X. (2006). Ultrasonic communication in frogs. Nature, 440(7082), 333–336. 10.1038/nature04416 16541072

[ece38854-bib-0017] Fontaine, S. S. , Novarro, A. J. , & Kohl, K. D. (2018). Environmental temperature alters the digestive performance and gut microbiota of a terrestrial amphibian. Journal of Experimental Biology, 221(20), jeb.187559. 10.1242/jeb.187559 30171093

[ece38854-bib-0018] Fu, H. B. , Zhang, L. Z. , Fan, C. , Li, W. J. , Liu, C. F. , Zhang, H. , Cheng, Q. , & Zhang, Y. M. (2021). Sympatric yaks and Plateau pikas promote microbial diversity and similarity by the mutual utilization of gut microbiota. Microorganisms, 9(9), 1890. 10.3390/microorganisms9091890 34576785 PMC8467723

[ece38854-bib-0019] Greer, R. L. , Dong, X. X. , Moraes, A. C. F. , Zielke, R. A. , Fernandes, G. R. , Peremyslova, E. , Vasquez‐Perez, S. , Schoenborn, A. A. , Gomes, E. P. , Pereira, A. C. , Ferreira, S. R. G. , Yao, M. , Fuss, I. J. , Strober, W. , Sikora, A. E. , Taylor, G. A. , Gulati, A. S. , Morgun, A. , & Shulzhenko, N. (2016). Akkermansia muciniphila mediates negative effects of IFN gamma on glucose metabolism. Nature Communications, 7, 13329. 10.1038/ncomms13329 PMC5114536 27841267

[ece38854-bib-0020] He, S. , Stevens, S. L. R. , Chan, L.‐K. , Bertilsson, S. , Glavina del Rio, T. , Tringe, S. G. , Malmstrom, R. R. , & McMahon, K. D. (2017). Ecophysiology of freshwater Verrucomicrobia inferred from Metagenome‐assembled genomes. mSphere, 2(5), e00277–17. 10.1128/mSphere.00277-17 28959738 PMC5615132

[ece38854-bib-0021] Huang, B. H. , Chang, C. W. , Huang, C. W. , Gao, J. , & Liao, P. C. (2018). Composition and functional specialists of the gut microbiota of frogs reflect habitat differences and agricultural activity. Frontiers in Microbiology, 8, 2670. 10.3389/fmicb.2017.02670 29375532 PMC5768659

[ece38854-bib-0022] Huang, G. P. , Wang, X. , Hu, Y. , Wu, Q. , Nie, Y. G. , Dong, J. H. , Ding, Y. , Yan, L. , & Wei, F. W. (2021). Diet drives convergent evolution of gut microbiomes in bamboo‐eating species. Science China Life Sciences, 64, 88–95. 10.1007/s11427-020-1750-7 32617829

[ece38854-bib-0023] Jing, T. Z. , Qi, F. H. , & Wang, Z. Y. (2020). Most dominant roles of insect gut bacteria: digestion, detoxification, or essential nutrient provision? Microbiome, 8(1), 38. 10.1186/s40168-020-00823-y 32178739 PMC7077154

[ece38854-bib-0024] Kanehisa, M. (2019). Toward understanding the origin and evolution of cellular organisms. Protein Science, 28(11), 1947–1951. 10.1002/pro.3715 31441146 PMC6798127

[ece38854-bib-0025] Kartzinel, T. R. , Hsing, J. C. , Musili, P. M. , Brown, B. R. P. , & Pringle, R. M. (2019). Covariation of diet and gut microbiome in African megafauna. Proceedings of the National Academy of Sciences of the United States of America, 116(47), 23588–23593. 10.1073/pnas.1905666116 31685619 PMC6876249

[ece38854-bib-0026] Kohl, K. D. , Cary, T. L. , Karasov, W. H. , & Dearing, M. D. (2013). Restructuring of the amphibian gut microbiota through metamorphosis. Environmental Microbiology Reports, 5(6), 899–903. 10.1111/1758-2229.12092 24249298

[ece38854-bib-0027] Langille, M. G. I. , Zaneveld, J. , Caporaso, J. G. , McDonald, D. , Knights, D. , Reyes, J. A. , Clemente, J. C. , Burkepile, D. E. , Thurber, R. L. V. , Knight, R. , Beiko, R. G. , & Huttenhower, C. (2013). Predictive functional profiling of microbial communities using 16S rRNA marker gene sequences. Nature Biotechnology, 31(9), 814–821. 10.1038/nbt.2676 PMC3819121 23975157

[ece38854-bib-0028] Li, J. , Ni, J. , Li, J. , Wang, C. , Li, X. , Wu, S. , Zhang, T. , Yu, Y. , & Yan, Q. (2014). Comparative study on gastrointestinal microbiota of eight fish species with different feeding habits. Journal of Applied Microbiology, 117(6), 1750–1760. 10.1111/jam.12663 25294734

[ece38854-bib-0029] Loudon, A. H. , Woodhams, D. C. , Parfrey, L. W. , Archer, H. , Knight, R. , McKenzie, V. , & Harris, R. N. (2014). Microbial community dynamics and effect of environmental microbial reservoirs on red‐backed salamanders (*Plethodon cinereus*). The ISME Journal Emultidisciplinary Journal of Microbial Ecology, 8(4), 830–840. 10.1038/ismej.2013.200 PMC3960541 24335825

[ece38854-bib-0030] Lupp, C. , Robertson, M. L. , Wickham, M. E. , Sekirov, I. , Champion, O. L. , Gaynor, E. C. , & Finlay, B. B. (2007). Host‐mediated inflammation disrupts the intestinal microbiota and promotes the overgrowth of Enterobacteriaceae. Cell Host & Microbe, 2(3), 204. 10.1016/j.chom.2007.06.010 18030708

[ece38854-bib-0031] Mohajeri, M. H. , Brummer, R. J. M. , Rastall, R. A. , Weersma, R. K. , Harmsen, H. J. M. , Faas, M. , & Eggersdorfer, M. (2018). The role of the microbiome for human health: from basic science to clinical applications. European Journal of Nutrition, 57(Suppl 1), 1–14. 10.1007/s00394-018-1703-4 PMC5962619 29748817

[ece38854-bib-0032] Montalban‐Arques, A. , Schryver, D. P. , Bossier, P. , Gorkiewicz, G. , Mulero, V. , Gatlin, D. M. , & Galindo‐Villegas, J. (2015). Selective manipulation of the gut microbiota improves immune status in vertebrates. Frontiers in Immunology, 6, 512. 10.3389/fimmu.2015.00512 26500650 PMC4598590

[ece38854-bib-0033] Petranka, J. W. (2007). Evolution of complex life cycles of amphibians: bridging the gap between metapopulation dynamics and life history evolution. Evolutionary Ecology, 21(6), 751–764. 10.3389/10.1007/s10682-006-9149-1

[ece38854-bib-0034] Qu, Y. F. , Wu, Y. Q. , Zhao, Y. T. , Lin, L. H. , Du, Y. , Li, P. , Li, H. , & Ji, X. (2020). The invasive red‐eared slider turtle is more successful than the native Chinese three‐keeled pond turtle: evidence from the gut microbiota. PeerJ, 8(12), e10271. 10.7717/peerj.10271 33194431 PMC7603792

[ece38854-bib-0035] R Development Core Team . (2020). R: a language and environment for statistical computing. R foundation for statistical computing. Available from: http://www.R‐project.org

[ece38854-bib-0036] Rojas, C. A. , Ramírez‐Barahona, S. , Holekamp, K. E. , & Theis, K. R. (2021). Host phylogeny and host ecology structure the mammalian gut microbiota at different taxonomic scales. Animal Microbiome, 3(1), 33. 10.1186/s42523-021-00094-4 33892813 PMC8063394

[ece38854-bib-0037] Rowland, I. , Gibson, G. , Heinken, A. , Scott, K. , Swann, J. , Thiele, I. , & Tuohy, K. (2018). Gut microbiota functions: metabolism of nutrients and other food components. European Journal of Nutrition, 57(1), 1–24. 10.1007/s00394-017-1445-8 PMC5847071 28393285

[ece38854-bib-0038] Schloss, P. D. , Westcott, S. L. , Ryabin, T. , Hall, J. R. , Hartmann, M. , Hollister, E. B. , Lesniewski, R. A. , Oakley, B. B. , Parks, D. H. , Robinson, C. J. , Sahl, J. W. , Stres, B. , Thallinger, G. G. , Van Horn, D. J. , & Weber, C. F. (2019). Introducing mothur: open‐source, platform‐independent, community‐supported software for describing and comparing microbial communities. Applied and Environmental Microbiology, 75(23), 7537–7541. 10.1128/AEM.01541-09 PMC2786419 19801464

[ece38854-bib-0039] Segata, N. , Izard, J. , Waldron, L. , Gevers, D. , Miropolsky, L. , Garrett, W. S. , & Huttenhower, C. (2011). Metagenomic biomarker discovery and explanation. Genome Biology, 12(6), R60. 10.1186/gb-2011-12-6-r60 21702898 PMC3218848

[ece38854-bib-0040] Shen, J. X. , Xu, Z. M. , Feng, A. S. , & Narins, P. M. (2011). Large odorous frogs (*Odorrana graminea*) produce ultrasonic calls. Journal of Comparative Physiology, A. Sensory, Neural, and Behavioral, Physiology, 197(10), 1027–1030. 10.1007/s00359-011-0660-7 21744010

[ece38854-bib-0041] Shu, Y. L. , Hong, P. , Tang, D. , Qing, H. , Donde, O. O. , Wang, H. , Xiao, B. D. , & Wu, H. L. (2019). Comparison of intestinal microbes in female and male Chinese concave‐eared frogs (*Odorrana tormota*) and effect of nematode infection on gut bacterial communities. MicrobiologyOpen, 8(6), e00749. 10.1002/mbo3.749 30311422 PMC6562124

[ece38854-bib-0042] Shu, Y. L. , Hong, P. , Yu, Q. , Wang, G. , Zhang, J. H. , Donde, O. , Xiao, B. D. , & Wu, H. L. (2019). High‐throughput sequencing analysis reveals correlations between host phylogeny, gut microbiota, and habitat of wild frogs from a mountainous area. Copeia, 107(1), 131–137. 10.1643/OT-18-040

[ece38854-bib-0043] Speakman, J. R. , Chi, Q. S. , Ołdakowski, Ł. , Fu, H. , Fletcher, Q. E. , Hambly, C. , Togo, J. , Liu, X. , Piertney, S. B. , Wang, X. , Zhang, L. , Redman, P. , Wang, L. , Tang, G. , Li, Y. , Cui, J. , Thomson, P. J. , Wang, Z. , Glover, P. , … Wang, D. (2021). Surviving winter on the Qinghai‐Tibetan Plateau: Pikas suppress energy demands and exploit yak feces to survive winter. Proceedings of the National Academy of Sciences of the United States of America, 118(30), e2100707118. 10.1073/pnas.2100707118 34282012 PMC8325355

[ece38854-bib-0044] Swidsinski, A. , Dörffel, Y. , Loening‐Baucke, V. , Theissig, F. , Rückert, J. C. , Ismail, M. , Rau, W. A. , Gaschler, D. , Weizenegger, M. , Kühn, S. , Schilling, J. , & Dörffel, W. V. (2011). Acute appendicitis is characterised by local invasion with Fusobacterium nucleatum/necrophorum. Gut, 60(1), 34–40. 10.1136/gut.2009.191320 19926616

[ece38854-bib-0045] Takei, Y. (2015). From aquatic to terrestrial life: evolution of the mechanisms for water acquisition. Zoological Society of Japan, 32(1), 1–7. 10.2108/zs140142 25660690

[ece38854-bib-0046] Tang, G. S. , Liang, X. X. , Yang, M. Y. , Wang, T. T. , Chen, J. P. , Du, W. G. , Li, H. , & Sun, B. J. (2020). Captivity influences gut microbiota in crocodile lizards (*Shinisaurus crocodilurus*). Frontiers in Microbiology, 11(550), 1–12. 10.3389/fmicb.2020.00550 32390955 PMC7190797

[ece38854-bib-0047] Tong, Q. , Cui, L. Y. , Du, X. P. , Hu, Z. F. , Bie, J. , Xiao, J. H. , Wang, H. B. , & Zhang, J. T. (2020). Comparison of gut microbiota diversity and predicted functions between healthy and diseased captive *Rana dybowskii* . Frontiers in Microbiology, 11(2096), 1–12. 10.3389/fmicb.2020.02096 32983063 PMC7490342

[ece38854-bib-0048] Tong, Q. , Du, X. P. , Hu, Z. F. , Cui, L. Y. , Bie, J. , Zhang, Q. Z. , Xiao, J. H. , Lin, Y. , & Wang, H. B. (2019). Comparison of the gut microbiota of *Rana amurensis* and *Rana dybowskii* under natural winter fasting conditions. FEMS Microbiology Letters, 366(21), fnz241. 10.1093/femsle/fnz241 31778183

[ece38854-bib-0049] Tong, Q. , Liu, X. N. , Hu, Z. F. , Ding, J. F. , Bie, J. , Wang, H. B. , & Zhang, J. T. (2019). Effects of Captivity and Season on the Gut Microbiota of the Brown Frog (*Rana dybowskii*). Frontiers in Microbiology, 10(1912), 1–12. 10.3389/fmicb.2019.01912 31507549 PMC6716059

[ece38854-bib-0050] Troyer, K. (1984). Behavioral acquisition of the hindgut fermentation system by hatchling *Iguana iguana* . Behavioral Ecology and Sociobiology, 14(3), 189–193. 10.1007/BF00299618

[ece38854-bib-0051] Vences, M. , Lyra, M. L. , Kueneman, J. G. , Bletz, M. C. , Archer, H. M. , Canitz, J. , Handreck, S. , Randrianiaina, R. D. , Struck, U. , Bhuju, S. , Jarek, M. , Geffers, R. , McKenzie, V. J. , Tebbe, C. C. , Haddad, C. F. B. , & Glos, J. (2016). Gut bacterial communities across tadpole ecomorphs in two diverse tropical anuran faunas. Science of Nature, 103(3), 25. 10.1007/s00114-016-1348-1 26924012

[ece38854-bib-0052] Videvall, E. , Song, S. J. , Bensch, H. M. , Strandh, M. , Engelbrecht, A. , Serfontein, N. , Hellgren, O. , Olivier, A. , Cloete, S. , Knight, R. , & Cornwallis, C. K. (2019). Major shifts in gut microbiota during development and its relationship to growth in ostriches. Molecular Ecology, 28(10), 2653–2667. 10.1111/mec.15087 30916826

[ece38854-bib-0053] Warne, R. W. , Kirschman, L. , & Zeglin, L. (2019). Manipulation of gut microbiota during critical developmental windows affects host physiological performance and disease susceptibility across ontogeny. Journal of Animal Ecology, 88(6), 845–856. 10.1111/1365-2656.12973 30828805

[ece38854-bib-0054] Weng, F. C. H. , Yang, Y. J. , & Wang, D. (2016). Functional analysis for gut microbes of the brown tree frog (*Polypedates megacephalus*) in artificial hibernation. BMC Genomics, 17(13), 1024. 10.1186/s12864-016-3318-6 28155661 PMC5260014

[ece38854-bib-0055] Wu, H. , Xing, Y. , Sun, H. , & Mao, X. (2019). Gut microbial diversity in two insectivorous bats: Insights into the effect of different sampling sources. MicrobiologyOpen, 8(4), e00670. 10.1002/mbo3.670 29971963 PMC6530527

[ece38854-bib-0056] Xu, L. L. , Chen, H. , Zhang, M. J. , Zhu, W. , Chang, Q. , Lu, G. Q. , Chen, Y. H. , Jiang, J. P. , & Zhu, L. F. (2020). Changes in the community structure of the symbiotic microbes in wild amphibians from the eastern edge of the Tibetan Plateau. MicrobiologyOpen, 9, e1004. 10.1002/mbo3.1004 32045512 PMC7142363

[ece38854-bib-0057] Ya, J. , Ju, Z. , Wang, H. , & Zhao, H. (2019). Exposure to cadmium induced gut histopathological damages and microbiota alterations of Chinese toad (*Bufo gargarizans*) larvae. Ecotoxicology Environmental Safety, 180, 449–456. 10.1016/j.ecoenv.2019.05.038 31121551

[ece38854-bib-0058] Zeevi, D. , Korem, T. , Godneva, A. , Bar, N. , Kurilshikov, A. , Lotan‐Pompan, M. , Weinberger, A. , Fu, J. Y. , Wijmenga, C. , Zhernakova, A. , & Segal, E. (2019). Structural variation in the gut microbiome associates with host health. Nature, 568(7750), 43–48. 10.1038/s41586-019-1065-y 30918406

[ece38854-bib-0059] Zhang, B. , Ren, J. , Yang, D. , Liu, S. , & Gong, X. (2019). Comparative analysis and characterization of the gut microbiota of four farmed snakes from southern China. Peer J, 7, e6658. 10.7717/peerj.6658 30956901 PMC6442672

[ece38854-bib-0060] Zhang, J. , Kobert, K. , Flouri, T. , & Stamatakis, A. (2014). PEAR: a fast and accurate Illumina Paired‐End reAd mergeR. Bioinformatics, 30(5), 614–620. 10.1093/bioinformatics/btt593 24142950 PMC3933873

[ece38854-bib-0061] Zhang, M. J. , Gaughan, S. , Chang, Q. , Chen, H. , Lu, G. Q. , Wang, X. G. , Xu, L. L. , Zhu, L. F. , & Jiang, J. P. (2019). Age‐related changes in the gut microbiota of the Chinese giant salamander (*Andrias davidianus*). MicrobiologyOpen, 8(7), e00778. 10.1002/mbo3.778 PMC6612560 30585426

[ece38854-bib-0062] Zhang, W. Y. , Li, N. , Tang, X. L. , Liu, N. F. , & Zhao, W. (2018). Changes in intestinal microbiota across an altitudinal gradient in the lizard *Phrynocephalus vlangalii* . Ecology and Evolution, 8(9), 4695–4703. 10.1002/ece3.4029 29760909 PMC5938461

[ece38854-bib-0063] Zhao, J. S. , Yao, Y. F. , Li, D. Y. , Xu, H. M. , Wu, J. Y. , Wen, A. X. , Xie, M. , Ni, Q. Y. , Zhang, M. W. , Peng, G. N. , & Xu, H. L. (2018). Characterization of the gut microbiota in six geographical populations of Chinese rhesus macaques (*Macaca mulatta*), implying an adaptation to high‐altitude environment. Microbial Ecology, 76, 565–577. 10.1007/s00248-018-1146-8 29372281

[ece38854-bib-0064] Zhou, J. , Zhao, Y. T. , Dai, Y. Y. , Jiang, Y. J. , Lin, L. H. , Li, H. , Li, P. , Qu, Y. F. , & Ji, X. (2020). Captivity affects diversity, abundance and functional pathways of gut microbiota in the northern grass lizard *Takydromus septentrionalis* . MicrobiologyOpen, 9(9), e1095. 10.1002/mbo3.1095 32666685 PMC7520994

[ece38854-bib-0065] Zhou, Y. , Shan, G. , Sodergren, E. , Weinstock, G. , Walker, W. A. , & Gregory, K. E. (2015). Longitudinal analysis of the premature infant intestinal microbiome prior to necrotizing enterocolitis: a case‐control study. PLoS One, 10(3), e0118632. 10.1371/journal.pone.0118632 25741698 PMC4351051

[ece38854-bib-0066] Zhu, L. , Liao, R. , Wu, N. , Zhu, G. , & Yang, C. (2019). Heat stress mediates changes in fecal microbiome and functional pathways of laying hens. Applied Microbiology and Biotechnology, 103(1), 461–472. 10.1007/s00253-018-9465-8 30368579

[ece38854-bib-0067] Zhu, L. F. , Zhang, Z. , Chen, H. , Lamer, J. T. , Wang, J. , Wei, W. Z. , Fu, L. X. , Tang, M. H. , Wang, C. H. , & Lu, G. Q. (2021). Gut microbiomes of bigheaded carps and hybrids provide insights into invasion: A hologenome perspective. Evolutionary Applications, 14(3), 735–745. 10.1111/eva.13152 33767748 PMC7980309

