# Erratum

**DOI:** 10.1002/ece3.8966

**Published:** 2022-05-24

In the recent article by Chen et al. (2022), the third institution for author Xiao‐Hong Chen should be removed. Her institutions should read as follows:

1. College of Life Sciences, Henan Normal University, Xinxiang, Henan, China.
2. The Observation and Research Field Station of Taihang Mountain Forest Ecosystems of Henan Province, Xinxiang, Henan, China.

We apologize for the inconvenience this may cause.
