# Supplementary figures and images for: Comparative study on gut microbiota in three Anura frogs from a mountain stream
Source: Ecol Evol. 2022 Apr 21;12(4):e8854. doi: 10.1002/ece3.8854 (PMC9021931; doi:10.1002/ece3.8854)

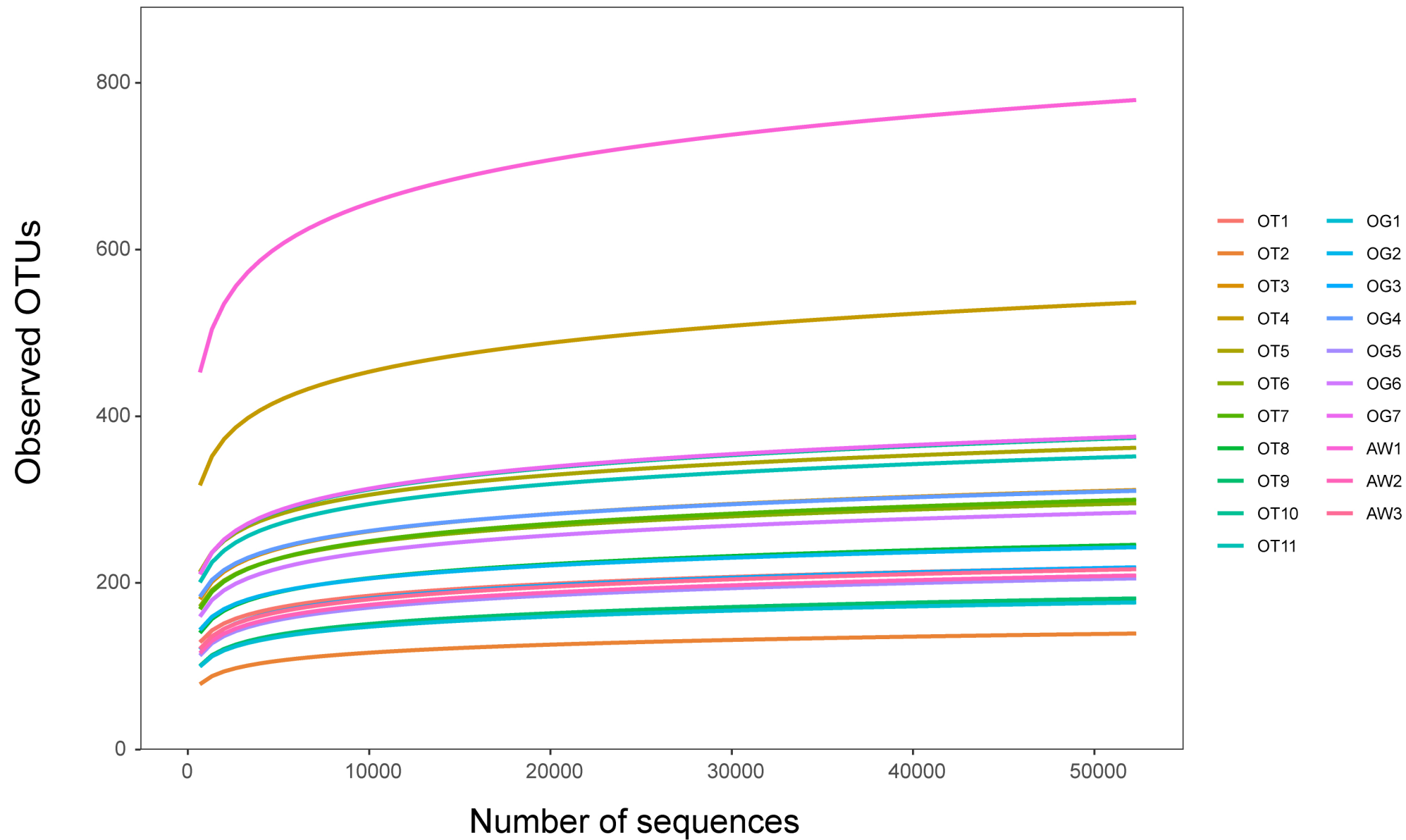

Supplement: Supplementary file 1 — Figure S1 [file ECE3-12-e8854-s002.pdf]
