# Supplementary material for: Comparative study on gut microbiota in three Anura frogs from a mountain stream
Source: Ecol Evol. 2022 Apr 21;12(4):e8854. doi: 10.1002/ece3.8854 (PMC9021931; doi:10.1002/ece3.8854)
Supplement: Supplementary file 2 — Table S1 [file ECE3-12-e8854-s001.docx]

Table S1 The number of operational taxonomic units (OTUs) and different bacterial taxonomic units of each sample.

| Sample ID | Species | OTUs | Genus | Family | Order | Class | Phylum |
| --- | --- | --- | --- | --- | --- | --- | --- |
| OT1 | *Odorrana tormota* | 213 | 100 | 57 | 38 | 19 | 12 |
| OT2 | *Odorrana tormota* | 145 | 65 | 34 | 21 | 13 | 8 |
| OT3 | *Odorrana tormota* | 310 | 100 | 51 | 29 | 14 | 8 |
| OT4 | *Odorrana tormota* | 524 | 147 | 63 | 40 | 17 | 9 |
| OT5 | *Odorrana tormota* | 358 | 98 | 54 | 31 | 16 | 9 |
| OT6 | *Odorrana tormota* | 294 | 115 | 61 | 39 | 15 | 8 |
| OT7 | *Odorrana tormota* | 308 | 97 | 54 | 35 | 19 | 10 |
| OT8 | *Odorrana tormota* | 248 | 106 | 59 | 40 | 19 | 10 |
| OT9 | *Odorrana tormota* | 189 | 68 | 43 | 29 | 15 | 9 |
| OT10 | *Odorrana tormota* | 382 | 120 | 59 | 41 | 23 | 12 |
| OT11 | *Odorrana tormota* | 354 | 116 | 64 | 41 | 21 | 11 |
| OG1 | *Odorrana graminea* | 180 | 70 | 46 | 31 | 19 | 11 |
| OG2 | *Odorrana graminea* | 239 | 99 | 57 | 36 | 18 | 10 |
| OG3 | *Odorrana graminea* | 228 | 66 | 42 | 24 | 15 | 8 |
| OG4 | *Odorrana graminea* | 303 | 105 | 57 | 34 | 21 | 11 |
| OG5 | *Odorrana graminea* | 216 | 95 | 51 | 34 | 18 | 12 |
| OG6 | *Odorrana graminea* | 294 | 105 | 62 | 40 | 22 | 13 |
| OG7 | *Odorrana graminea* | 386 | 124 | 65 | 45 | 26 | 12 |
| AW1 | *Amolops wuyiensis* | 775 | 185 | 77 | 44 | 20 | 11 |
| AW2 | *Amolops wuyiensis* | 217 | 102 | 63 | 37 | 22 | 14 |
| AW3 | *Amolops wuyiensis* | 223 | 102 | 65 | 45 | 24 | 12 |
